# Processing Load Induced by Informational Masking Is Related to Linguistic Abilities

**DOI:** 10.1155/2012/865731

**Published:** 2012-10-03

**Authors:** Thomas Koelewijn, Adriana A. Zekveld, Joost M. Festen, Jerker Rönnberg, Sophia E. Kramer

**Affiliations:** ^1^Department of ENT/Audiology and EMGO Institute for Health and Care Research, VU University Medical Center, Boelelaan 1117, 1081 HV Amsterdam, The Netherlands; ^2^Linnaeus Centre HEAD, Swedish Institute for Disability Research, Linköping University, 581 83 Linköping, Sweden; ^3^Department of Behavioral Sciences and Learning, Linköping University, 581 83 Linköping, Sweden

## Abstract

It is often assumed that the benefit of hearing aids is not primarily reflected in better speech performance, but that it is reflected in less effortful listening in the aided than in the unaided condition. Before being able to assess such a hearing aid benefit the present study examined how processing load while listening to masked speech relates to inter-individual differences in cognitive abilities relevant for language processing. Pupil dilation was measured in thirty-two normal hearing participants while listening to sentences masked by fluctuating noise or interfering speech at either 50% and 84% intelligibility. Additionally, working memory capacity, inhibition of irrelevant information, and written text reception was tested. Pupil responses were larger during interfering speech as compared to fluctuating noise. This effect was independent of intelligibility level. Regression analysis revealed that high working memory capacity, better inhibition, and better text reception were related to better speech reception thresholds. Apart from a positive relation to speech recognition, better inhibition and better text reception are also positively related to larger pupil dilation in the single-talker masker conditions. We conclude that better cognitive abilities not only relate to better speech perception, but also partly explain higher processing load in complex listening conditions.

## 1. Introduction

A major complaint of both hearing-impaired and normal hearing individuals is the high level of effort while following a conversation in a noisy situation. Although sensory hearing loss is considered the main cause of speech communication difficulties [1, 2], comprehension of speech in noise is not fully predicted by a pure-tone audiogram or other psycho-acoustical tests [3–6]. Research has shown that speech comprehension and related listening effort are not only based on sensory processes, but also on linguistic and working-memory-related cognitive abilities [2, 7, 8]. These insights were obtained as the result of two major areas of science (namely, hearing sciences and cognitive sciences) merging into one area of cognitive hearing science (CHS) which we witnessed during the last decade [9]. A next step in CHS research would be the examination of the interaction between use and benefit of devices like hearing aids, and individuals' cognitive abilities and mental effort [10, 11]. Attempts into that direction were made by Gatehouse et al. [12, 13] who observed a relationship between an individual's cognitive abilities and candidature for a certain hearing aid fitting pattern. However, before these and other insights obtained within CHS can be applied to clinical practice (i.e., hearing aid fitting evaluation) we need to know more precisely what cognitive processes is associated with listening effort. Although it is often assumed that the involvement of cognitive functions in speech comprehension is responsible for the listening effort that people experience, it is not known yet how these two are related. In other words: how do cognitive factors differentially impact on (a) speech understanding and (b) effort or processing load deployed during listening. This is what we focused on in the current study. We investigated how cognitive capacity associates to speech comprehension and relates to listening effort in normal hearing participants. 

In our recent study [14] we found that cognitive load during speech processing differed for different types of background noise. It was observed that listening to speech masked by a single talker evoked a larger pupil dilation response than listening to speech masked by fluctuating or stationary noise. The effect was independent of speech intelligibility level. The authors concluded that the effect was most likely caused by semantic interference of the single talker masker. Cognitive abilities supposedly associated with this effect are working memory (WM) [8, 15, 16], the ability to inhibit irrelevant speech while storing information in WM [17, 18], and linguistic abilities [3, 19, 20]. Note that the inhibition of irrelevant speech information is an important capacity in daily life listening. While listening to a speaker, listeners often have to neglect other irrelevant speakers (e.g., during a cocktail party) [21]. The inhibition of information could be one of the cognitive functions affecting processing load during listening. 

Whereas the role of these abilities in speech *perception* has been repeatedly demonstrated, their relation with the *processing load* evoked by speech perception, as assessed by pupillometry, has been examined rarely. One of the studies in which this issue was explored was Zekveld et al. [22]. However, Zekveld et al. did not measure working memory capacity and neither included different types of noise maskers. The aim of the current study was to investigate the relation of cognitive abilities and the processing load induced by perceiving speech in different types of distracter conditions. Specifically, the study aimed to address the associations between these cognitive abilities during listening and the additional load imposed by the semantic interference of a single-talker masker as compared to the masking imposed by a fluctuating noise masker [14].

Processing load is shown to be reflected by pupil dilation as measured with pupillometry [23, 24]. Pupil dilation is also related to effort caused by allocation of attentional resources [25]. Interestingly, pupil dilation is sensitive to language processing tasks like hearing and reading words or sentences [26–30]. In a pioneering study, Kramer et al. [28] investigated the pupil response in relation to speech processing in adverse listening conditions. By using pupillometry in combination with a speech reception threshold (SRT) task, it was shown that the SNR affected processing load as reflected by changes in the pupil dilation response (see also, [22, 31]). 

Zekveld et al. [22] showed that subjects with better text reception thresholds (TRT) allocated more cognitive resources (larger pupil dilations) during speech perception in stationary noise. This effect was independent of intelligibility level. Additionally, Zekveld et al. [32] observed that better TRT performance was associated with increased brain activation in the left angular gyrus (AG) during cued speech perception. AG is associated with “combinatorial semantic processing” [33] a process in which word fragments are combined into full sentences by means of semantic structure. These results may suggest that individuals with good TRT performance employ a different strategy during listening that may require more processing load. However, we have to be careful in directly relating these results on AG activation with pupillometry data as obtained in the current study, because the pupil response reflects a more co

mplex network of brain areas than AG alone [34]. Contrasting findings were observed for RSpan capacity. Larger RSpan capacity was associated with *less* activation in inferior frontal and superior temporal brain regions. To gain more insight into the factors involved in this complex area of research, we assessed both WMC and TRT in the current study to investigate how interindividual differences in these measures affect SRT and pupil dilation. 

In the present study, we included several measures of cognitive ability known to be related to speech comprehension and often assumed to be related to processing load during listening. First, we assessed WM capacity (WMC). According to the “ease of language understanding” (ELU) model [35], WM is strongly involved in language processing specifically when speech is partly masked by fluctuating background sound [8, 16, 17, 36]. In such challenging situations, WM enables the listener to keep a mental representation of a spoken sentence while using knowledge of language and context to fill in gaps in the information. WMC has often been shown to partly explain the frequently observed interindividual differences in speech processing in adverse listening conditions [15, 35–37]. 

Therefore, in the current study we applied a Dutch version of the reading span (Rspan) as well as a listening span (Lspan) task. Both tests are adaptations of the Rspan test used in previous studies [38–41]. Secondly, a Dutch version of the size-comparison span (SICspan) task was included [17, 18]. The SICspan measures the ability to inhibit irrelevant linguistic information while storing information in WM. Better inhibition of the distractor items in addition to remembering more target items will lead to a higher SICspan score. Finally, we included the TRT test [20], assessing combinatorial semantic processing [32] by asking subjects to read partly masked text. This ability is associated with both speech perception in noise [7, 42] and the cognitive processing load during listening, as indicated by both pupillometric [22] and functional magnetic resonance imaging (fMRI) data [32]. Note, that the TRT is not a measure of cognitive capacity like the span tasks. Instead it is a visual equivalent of the SRT, a threshold of language comprehension in adverse conditions. In the current study, we investigated the differential effect of an individual's cognitive abilities on listening effort and speech understanding in normal hearing adults. This knowledge is required when considering the method of pupillometry for use in hearing aid fitting evaluations in the future. Participants performed an SRT task in fluctuating noise and against the background of a single-talker masker. The adaptive procedures targeted either 50% or 84% correctly repeated sentences. During the tasks, pupil responses were recorded and subjective effort, performance, and motivation ratings were acquired at the end of each block of sentences. The primary aim of the study was to examine if the pupil response evoked by semantic interference was related to WMC, WM-related inhibition, or TRT. We examined the associations between the peak pupil dilation (PPD) and individuals' linguistic and cognitive abilities. Since we observed larger pupil responses for speech masked by an interfering talker as compared to speech masked by a fluctuating masker in our previous study, we also examined the differences in PPD between fluctuating noise and a single-talker masker conditions. Based on previous studies that already investigated some of these effects and associations [14, 22, 32], we expected that a higher cognitive capacity would be associated with a larger pupil response during speech perception in more complex conditions compared to easy listening conditions. 

## 2. Methods

### 2.1. Participants

Thirty-two adults (aged between 40 and 70 years, mean age 51.3 years, 6 males) with normal hearing, recruited at the VU University Medical Centre, participated in the study. Normal hearing was defined as having pure-tone thresholds less than or equal to 20 dB HL at the individual frequencies 250, 500, 1000, 2000, and 4000 Hz in both ears, having no more than a single 35 HL dB dip at one of these frequencies in one ear. Participants had no history of neurological diseases, reported normal or corrected-to-normal vision, and were screened for near-vision acuity [43]. They were native Dutch speakers and provided written-informed consent in accordance with the Ethics Committee of the VU University Medical Center. 

### 2.2. SRT

The speech reception threshold (SRT) [44] was measured by presenting speech in fluctuating noise or masked with a single-talker masker [14, 45]. The SRT adaptively assessed the SNR required to perceive either 50% or 84% of the sentences entirely correctly [44, 46]. Both masker types had a long-term average spectrum adapted to the spectrum of the target speech signal [47]. The target sentences were spoken by a female voice and for the single-talker masker concatenated sentences were used, spoken by a male voice with modified spectrum. The fluctuating noise mimicked the intensity fluctuations of speech, by multiplying the noise signal by the envelope of the speech of the single-talker masker. Each of these four conditions was measured in a blocked fashion and the level of the target speech was fixed at 55 dBA. Each block contained 39 short Dutch sentences [47] and the order of the blocks was counterbalanced over participants. 

### 2.3. TRT

The text reception threshold (TRT) task [20] is a visual analog to the SRT task. In this task participants read text sentences presented on a computer screen in red font on a white background and masked by black vertical bars. Sentences appeared on a screen word by word with a similar timing as the word onsets in the corresponding recorded SRT sentences. After the onset of the last word, the full sentences remained on the screen for 500 ms [42]. A 1-up-1-down adaptive procedure with a step size of 6% was applied, targeting the percentage of unmasked text required to read 50% of the sentences entirely correctly. The sentences presented were selected from the same corpus as used for the SRT [47] but did not overlap with those presented in the SRT tests. Participants performed four tests with 13 sentences each; the first test was a practice test of which the data were excluded from the analysis. The TRT was the average percentage of unmasked text in the three remaining tests with the first four sentences omitted. Lower thresholds indicate better performance.

### 2.4. Rspan and Lspan

Reading span (Rspan) and listening span (Lspan) tests were used to assess verbal WM capacity in the visual and auditory domain, respectively. Each test consisted of 54 sentences that were presented in sets of 3 to 6 sentences. Half of the sentences were semantically incorrect. Participants did not know beforehand whether they were to remember and report the initial or final noun of each sentence. After the presentation of each block, they had to repeat either the last or first words in the correct order. This kind of postcueing procedure makes the task less strategically manageable and hence more difficult. In addition, participants performed a semantic judgment task after each individual sentence during presentation of the sentence set. In the Rspan test each sentence was visually presented [42, 48]. The Lspan sentences were presented dichotically through headphones at 65 dBA. Subjects responded verbally. Prior to each test participants practiced on 10 sentences divided over three sets. The span size corresponds to the total number of correctly recalled target words irrespective of their order of presentation, with a maximum score of 54. Higher scores indicate better performance. 

### 2.5. SICspan

In the “size-comparison span” (SICspan) task [17, 18], participants were asked to make relative size judgments between two items (e.g., Is LAKE bigger than SEA?) by pressing “J” key for yes and “N” for no on a QWERTY keyboard. Each question was followed by a single to-be-remembered item, which was semantically related to the objects in the sentence (e.g., RIVER). Ten sets were presented: 2 to 6 size comparison questions each, followed by a to-be-remembered item. Participants were asked to verbally recall the to-be-remembered items in order of presentation. Sentences and to-be-remembered items within each set were from the same semantic category, but between sets, the semantic categories were different. The SICspan score used in this study contained all correctly remembered items independent of order, which leads to a maximum score of 40. The higher the score the better the performance on the SICspan task.

### 2.6. Apparatus

Participants were tested in a sound-treated room. During the SRT task participants had to fixate their gaze to a dot (diameter 0.47°) located at 3.5 meter distance, at eye level on a white wall. Throughout the SRT test, the pupil diameter of the left eye was measured by an infrared eye tracker (SMI, 2D Video-Oculography, version 4). Light intensity was adjusted by an overhead light source so that the pupil diameter was around the middle of its dilation range at the start of the experiment. For both the SRT and the Lspan task, audio in the form of separate files (44.1 Hz, 16 bit) was presented binaurally by an external soundcard (Creative Sound Blaster, 24 bit) through headphones (Sennheisser, HD 280, 64 Ω). During all visual tasks and during the Lspan task participants were facing a computer screen (Dell, 17 inch) at 60 cm distance. All tests were presented by a Windows PC (Dell, Optiplex GX745, 2.66 GHz 2 Core).

### 2.7. Procedure

Participants started the test session with either the Rspan or the Lspan task (order was balanced over subjects). Additionally, they performed two-blocked conditions of the SRT task (order was balanced over subjects). After a 10-minute break participants performed the SICspan task followed by the two remaining experimental conditions of the SRT task. After a second 10-minute break participants performed the remaining Lspan or Rspan task. Participants ended the session by performing the TRT task. The test session took 2.5 to 3 hours. 

During the SRT task the pupil response was used as a measure of processing load. Pupil traces and SRT data of the trials containing the first four sentences were omitted further from analysis. For all remaining traces diameter values more than 3 SDs smaller than the mean were coded as blinks. Traces containing more than 15% blinks were excluded others were deblinked by means of a linear interpolation. A spike detection algorithm was used to detect eye movements (for a full description see, [49]) on both the *x*- and *y*-traces. All trials with a range in *x*- or *y*-amplitudes exceeding 2 SDs, within a sliding window of 100 ms, were excluded from analysis. All remaining traces were baseline corrected by subtracting the mean pupil size within the 1-second period prior to the speech onset. The PPD was calculated for each subject for each condition. PPD was defined as the highest value within a time window of 4.4 seconds after speech onset, which resembled the interval between speech onset and the response prompt. 

After each SRT block, participants rated their effort, performance, and motivation level during the block. Participants had to indicate how much effort it took to perform the SRT task on a continuous scale from 0 (“no effort”) to 10 (“very effortful”). Additionally, participants indicated how they themselves perceived their performance on the task by rating between 0 (“none of the sentences were intelligible”) and 10 (“all sentences were intelligible”). Finally, to assess their degree of motivation during the course of the test, participants indicated how often during the block they had abandoned the listening task, because the task was too difficult. This was rated between 0 (“this happened for none of the sentences”) and 10 (“this happened for all of the sentences”). Prior to analysis the motivation score was inversed, so high scores reflect high motivation. Note that a continuous scale (range between 0–10) was applied. Participants were explicitly instructed that they could give ratings in between the whole numbers on the scale, which was reflected in the raw scores that also showed a normal distribution when tested for skewness and kurtosis. For more details on stimuli, SRT procedure, pupillometry, and subjective ratings see Koelewijn et al. [14].

### 2.8. Statistical Analysis

A repeated measures analysis of variance (ANOVA) testing the effects of intelligibility (50% and 84%) and masker type (fluctuating noise and single-talker masker) was performed on the SRT scores, PPD, and the subjective ratings. Statistically significant (*P* < .05) interactions were further analyzed by means of two-tailed paired samples *t*-tests. Additionally, Pearson correlation coefficients were calculated to test the relations between age, PTA, Rspan, Lspan, SICspan, TRT, and SRT. Finally, linear regression analyses were performed to examine the associations between interindividual differences in SRT or PPD (dependent variables) and cognitive abilities (Rspan, Lspan, SICspan) and TRT as independent factors. For each dependent variable, regression analyses were performed separately for each masker type and both intelligibility levels. 

In addition, we used regression models to examine the associations between cognitive abilities, and the additional PPD imposed by semantic interference. The same was done for the association between TRT and this informational masking effect. We calculated difference scores for both the SRT (Δ_SRT_) and PPD (Δ_PPD_) by subtracting the outcome for fluctuating noise averaged over both intelligibility conditions from the outcome for the single talker averaged over both intelligibility conditions. For each of the regression models, we examined whether age and PTA were each individually confounding the relationship between the dependent and independent variables. A variable was considered as a relevant confounder when the regression coefficient changed with 10% or more after adding the potential confounder to the analysis. Additionally, the potential confounder had to be associated with both the independent (cognitive abilities and TRT) and the dependant (SRT or pupil response) factors. All statistical analyses were performed using SPSS version 17. 

## 3. Results

### 3.1. Behavioral Results SRT

**Figure 1:**
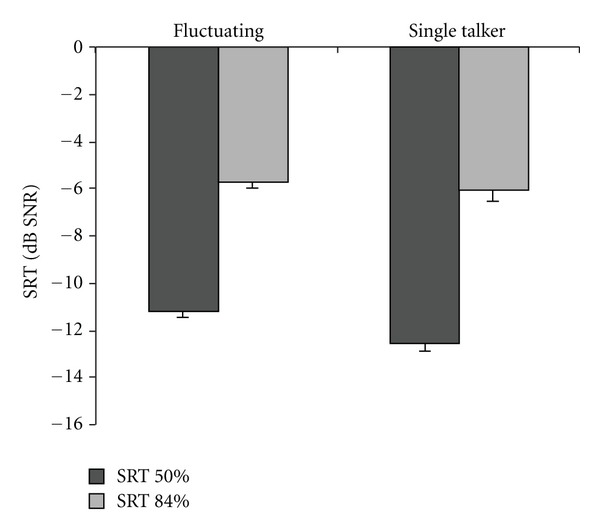
SRTs (dB SNR) at two intelligibility levels for both masker types, averaged over subjects. The error bars show the standard errors for each condition.

The average SRTs (dB SNR) in fluctuating noise and in the single-talker masker, at intelligibility levels of 50% and 84%, are plotted in Figure 1. The average SRT, PPD, and the subjective ratings for each condition are reported in Table 1.

An ANOVA on the SRTs showed a main effect of intelligibility (*F*_[1,31]_ = 438.82, *P* < .001) with a lower SRT_50%_ (mean SNR = −11.9 dB) compared to SRT_84%_ threshold (SNR = −5.9). Additionally, a main effect of masker type was observed (*F*_[1,31]_ = 5.09, *P* = .031) showing a slightly lower threshold for the single-talker masker (SNR = −9.3) than in fluctuating noise (SNR = −8.5). Also, an interaction between intelligibility level and masker type was observed (*F*_[1,31]_ = 5.66, *P* = .024). Post hoc analysis revealed a significant difference at 50% intelligibility between the single-talker masker (SNR = −12.6) and fluctuating noise (SNR = −11.2) conditions (*t*_[31]_ = −3.65, *P* = .001), and no masker effect at the 84% intelligibility level. These results indicate an overall effect of intelligibility level on the SRT and a slightly lower SRT in single talker compared to fluctuating noise at 50% intelligibility.

### 3.2. Pupil Data SRT

**Figure 2:**
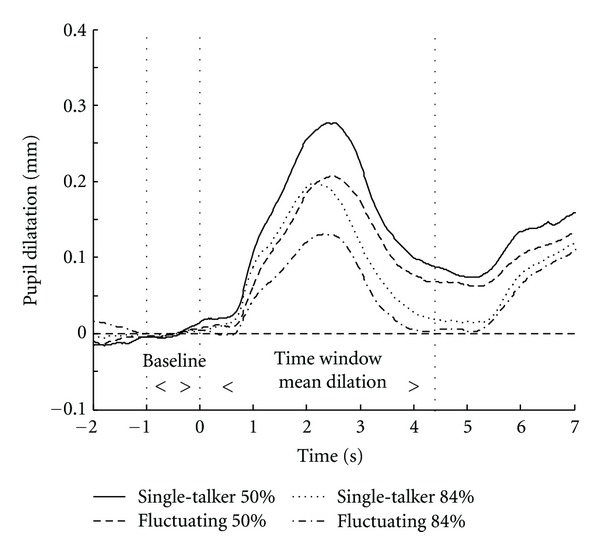
Pupil responses per condition averaged over subjects. The onset of the sentences is at 0 sec. The baseline is indicated as the average pupil diameter over one second preceding the start of the sentence. The area between the second and third dotted lines indicates the time window used for calculating the mean pupil dilation.

Pupil traces containing a large number of blinks (in total 6.0% of the traces) and/or large eye movements (in total 9.8% of the traces) were removed from further analysis. PPD was calculated over the remaining traces for each condition. The average traces for the four conditions are plotted in Figure 2. 

An ANOVA on PPD revealed a main effect of intelligibility level (*F*_[1,31]_ = 20.31, *P* < .001), with a larger PPD in the SRT_50%_ conditions (0.26 mm) compared to the SRT_84%_ conditions (0.19 mm). Additionally, there was an effect of masker type (*F*_[1,31]_ = 40.66, *P* < .001) with a larger average PPD for the single-talker masker (0.26 mm) compared to fluctuating noise (0.20 mm). No interaction between intelligibility level and masker type was observed (*F*_[1,31]_< 1).

### 3.3. Subjective Ratings SRT

An ANOVA was performed for each of the three subjective ratings separately (Table 1). An effect of intelligibility level on the *subjective effort* ratings was observed (*F*_[1,31]_ = 46.50, *P* < .001), indicating that subjectively, lower intelligibility makes speech perception more effortful. Masker type did not affect the ratings (*F*_[1,31]_ < 1). However, an interaction between intelligibility level and masker type was observed (*F*_[1,31]_ = 6.45, *P* = .016). Post hoc analysis revealed that only in the 84% condition, a significant difference in subjective effort between the single-talker masker (5.3) and fluctuating noise (4.7) conditions was found (*t*_[31]_ = −2,18, *P* = .037). Note that the 84% condition SRTs for fluctuating noise (SNR = −5.7) and the single-talker masker (SNR = −6.0) did not differ significantly (*t*_[31]_ < 1). An effect of intelligibility on the *subjective performance *ratings was observed (*F*_[1,31]_ = 105.12, *P* < .001) showing lower ratings at 50% intelligibility (5.5) then at 84% intelligibility (7.0). Additionally, no effect of masker type (*F*_[1,31]_ < 1) or interaction effect (*F*_[1,31]_ < 1) was observed. *Subjective motivation *ratings showed a main effect of intelligibility level (*F*_[1,31]_ = 19.77, *P* < .001). No effect of masker type (*F*_[1,31]_ < 1) or interaction (*F*_[1,31]_ < 1) was observed. Participants were less motivated in the 50% intelligibility conditions (7.9) compared to the 84% conditions (8.6). 

### 3.4. Descriptive Statistics Cognitive Tests

For each subject we calculated the total scores for the Rspan (mean = 15.5, sD = 4.4), Lspan (mean = 21.4, sD = 3.6), and SICspan (mean = 23.8, sD = 6.1). Additionally, the individual TRTs were calculated (mean = 59.8, sD = 5.5). Correlation analyses showed that there were no significant correlations between age and each of the span tasks (Rspan, Lspan, and SICspan), or age and TRT. Pearson correlations between each of the cognitive tests (Rspan, Lspan, SICspan) and TRT ranged between .58 and .77 and were statistically significant (Table 2). 

### 3.5. Relation between Cognitive Abilities, Speech Perception, and Processing Load

 To examine whether SRT and PPD during speech perception were associated to WMC (Rspan, Lspan), inhibition (SICspan), and linguistic processing (TRT), regression analyses were performed for the behavioral and PPDs separately. The slope (*B*), the variance (*R* ^2^), and the *P* values for the independent factors explaining the performance in SRT_50%_ and SRT_84%_ are shown in Table 3(a). Table 3(b) shows the results for the PPD in SRT_50%_ and SRT_84%_. In none of the equations, PTA was a confounder and hence, not adjusted. Age appeared to be a confounder in some of the analyses, in which case age was included in the model. Only significant (*P* < .05) associations are shown. Note that separate models were run for each of the cognitive measures, to eliminate colinearity. Analyzing each of the cognitive functions separately allowed us to examine and compare the individual association with the dependent variables. Note that the reported *R* ^2^ is always based on the single-dependent measure. 

The outcomes for the SRT regression analyses (Table 3(a)) showed no significant associations for the fluctuating noise condition at 50% intelligibility (SRT_F50_). For the fluctuating noise condition at 84% intelligibility (SRT_F84_), a significant association with Rspan and SICspan was found. For the single-talker masker at 50% (SRT_ST50_) and 84% intelligibility (SRT_ST84_), significant associations were found for Rspan, SICspan, and TRT. In all models, higher (better) Rspan and SICspan scores were related to lower (better) SRTs. Additionally, lower (better) TRTs were related to lower (better) SRTs. 

 The outcomes for the regression analyses with PPD as the dependent measure (Table 3(b)) showed no associations between PPD and cognitive abilities in both fluctuating noise conditions. However, in the single-talker masker condition at 50% intelligibility, the PPD (PPD_ST50_) was significantly associated with SICspan and TRT, and PPD for the single-talker masker at 84% intelligibility (PPD_ST84_) was associated with SICspan. In these associations, higher SICspan scores related to a larger PPD and lower (better) TRTs also related to a larger PPD. 

**Figure 3:**
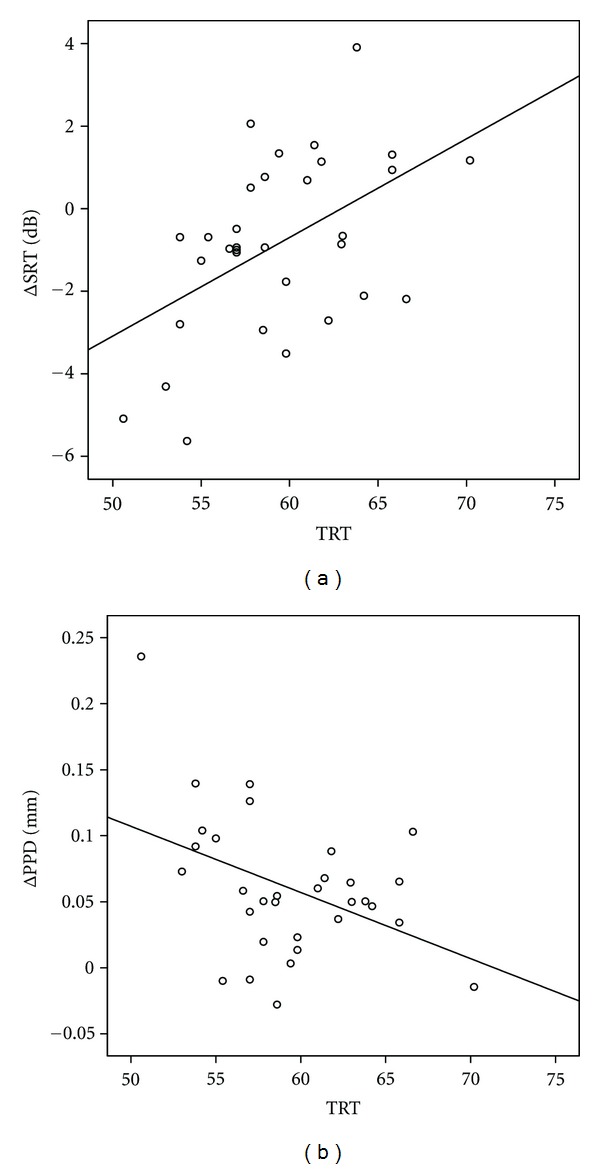
(a) TRT performance as function of ΔSRT [(ST50 + ST84/2)−(F50 + F84/2)]. (b) TRT as function of ΔPPD.

The main question of this study was which cognitive abilities are associated with the performance benefit and the additional processing load imposed by semantic interference? To answer this question, we performed regression analyses with similar independent variables as before, but now with Δ_SRT_ and Δ_PPD_ as dependent variables (Table 4). The variance in Δ_SRT_ was significantly associated with Rspan (*R* ^2^ = .14, *P* = .035) and TRT (*R* ^2^ = .25, *P* < .01). However, after correcting for age, the association with Rspan was no longer significant (*P* = .085). Lower (better) TRTs were associated with negative Δ_SRT_ scores. These negative scores occurred when participants performed better in the single-talker conditions than in the fluctuating noise conditions. Higher (poorer) TRTs were associated with positive Δ_SRT_ scores. These positive scores occurred when participants performed better in the fluctuating noise conditions than in the single-talker conditions. Only the TRT explained part of the variance in Δ_PPD_ (*R* ^2^ = .176, *P* = .017), with better TRTs associated with a larger difference in processing load between the two masker types. Scatterplots of the significant associations between TRT, and the difference scores Δ_SRT_ and Δ_PPD_ are shown in Figures 3(a) and 3(b).

Finally, to investigate whether the SRT and PPD were independent measures we calculated the Pearson correlations between SRT and PPD for all four conditions. For both the fluctuating noise 50% and 84% intelligibility conditions, no significant correlations were found. For the single-talker masker conditions we found a significant correlation in the 84% intelligibility condition (*r*_s_ = −0.594, *P* < .01). The negative correlations indicated that a lower (i.e., better) SRT was related to larger pupil dilation. 

### 3.6. Relation between Subjective Ratings and PPD

We calculated Spearman correlation coefficients between the PPD and subjective ratings for each of the four SRT conditions. A Spearman correction was used to account for the skewed distribution of the subjective ratings. *Subjective effort* was only significantly correlated with the PPD in fluctuating noise at 84% intelligibility (*r*_s_ = 0.428, *P* < .05), with larger subjective effort associated with larger PPDs. *Subjective performance* ratings correlated significantly with PPD for fluctuating noise at 50% intelligibility (*r*_s_ = −0.486, *P* < .01) and for the single-talker masker condition at 84% intelligibility (*r*_s_ = −0.386, *P* < .05). Subjects who indicated that they had relatively high performance levels had low processing load as indicated by the PPD. Subjective motivation ratings did not significantly correlate with PPD.

## 4. Discussion

This study aimed to address the associations between cognitive abilities and the additional load imposed by the semantic interference during speech perception. In line with our previous study [14], we observed that the pupil response was larger in the single-talker masker conditions than in the fluctuating noise conditions. These findings reflect increased processing load evoked by semantic interference during the perception of speech, independent of intelligibility level. These results were not shown by the traditional SRT data and only partly by the subjective effort ratings. This clearly supports the advantage of the application of pupillometry over performance measures and subjective ratings. 

The novel finding of the current study is that the additional processing load (Δ_PPD_) due to semantic interference is associated with interindividual differences in written text reception (TRT), as shown in the regression models (Tables 3(a) and 3(b)). Additionally, we found larger PPDs in the single-talker conditions to be associated to better SICspan scores. TRT contributed significantly in the regression model explaining PPD in the single-talker masker condition at 50% intelligibility level. The association was such that better performance on the TRT was related to a larger PPD. This is in line with Zekveld et al. [22]. Apparently, the abilities captured by this TRT test are relevant to the perception of speech when masked by interfering speech as compared to fluctuating noise. In order for these abilities to be involved in both auditory and written language processing, they most likely occur at a modality-independent level [50]. Therefore, these outcomes suggest the involvement of higher amodal cognitive processes in the comprehension of speech when masked by an interfering talker. The current findings seem to agree with Zekveld et al. [32] who showed more activation in the angular gyrus in individuals with better TRTs.

 The SICspan was related to the PPDs for the single-talker masker at both intelligibility levels, such that a higher capacity was associated with larger PPDs. This is opposite to the other research that shows higher WMC in association with a *smaller* pupil size (e.g., [51]). Note that although the Rspan and SICspan both assess WMC, the SICspan additionally reflects a person's ability to inhibit irrelevant linguistic information [17]. These results thereby may suggest that processing load, and the way the brain deals with it—as reflected by PPDs—is predominantly related to active inhibition of irrelevant linguistic information rather than storage capacity per se. This is in line with the idea of Kahneman [25] that the pupil response reflects attention. Attending to relevant information during speech processing when there is interfering speech seems to be reflected by the PPDs.

The Rspan, SICspan, and TRT explained a substantial part of the variation in the SRT scores. Better TRTs were associated with lower SRT-advantage in a single-talker masker over a fluctuating noise (negative ΔSRT in Figure 3(a)). However, poorer TRTs were associated with an SRT-disadvantage in a single-talker masker over a fluctuating noise (positive ΔSRT Figure 3(a)). Additionally, current results show an association between Rspan and the SRTs obtained the single-talker conditions. The findings agree with Besser et al. [42] who found that the TRT and Rspan are capturing different aspects of speech perception. A better ability to inhibit semantically related items, as indicated by the SICspan was associated with lower SRTs suggesting a role of this function in processes that aid perception. The results suggest a stronger involvement of higher cognitive processes during the single-talker masker conditions in comparison to fluctuating noise.

 Although highly correlated to the Rspan task, the Lspan did not explain any of the effects. The results showed that participants scored significantly higher on the Lspan. There was less variance in the Lspan scores compared to the Rspan scores. This suggests that the Lspan task was easier than the Rspan and might explain the lack of explained variance in the criterion variables. 

We did not find any confounding effects of PTA, which is not surprising when testing a group of normal hearing people. Age however was a significant confounder in some of the regression models. Age is known to have an effect on speech perception (e.g., [3, 52]), which was clearly reflected by the correlations between age and the SRTs in the single-talker conditions as shown in Table 2. Our results confirmed that SRTs increased (worsened) with age.

It might be argued as counterintuitive that better cognitive abilities evoke larger processing load (PPD) during listening to speech in noise. However, this relation is twofold. First, the relation between SRT and cognitive capacity showed a clear performance “benefit” for people with a higher capacity, since those with a better SICspan perform better on speech intelligibility in noise. Second, the deployment of this higher capacity comes with the “cost” of slightly more “cognitive load”, or more extensive/intensive use of the brain, in the more difficult listening conditions. Note that the significant contribution of the cognitive variables in the models explaining PPD, the cognitive variables only explained a “small” part of the variance as shown by the *R* ^2^ values in Table 3(b), compared to the variance explained for the SRT's (performance). Although these associations are small, they do suggest involvement of higher cognitive processes in listening effort. 

Remarkably, the effect of the single-talker masker on PPD was independent of intelligibility level. This is a little surprising when considering 84% as less challenging than 50% intelligibility. However, the single-talker masker in this study was always presented at an audible level and therefore semantic interference could occur independent of target speech intelligibility. Additionally, 50% and 84% intelligibility may still be considered within the doable range and not be considered very difficult or very easy. Therefore, tests at a broader range of intelligibility levels might be required in order to observe interactions between intelligibility and semantic interference. 

Subjective effort ratings correlated with load as shown by the PPD, but only for fluctuating noise at high intelligibility (i.e., 84%). Additionally, subjective performance ratings correlated with PPD as well for fluctuating noise at low intelligibility and for the single talker at high intelligibility. Although intelligibility levels were kept constant, people tend to under-or overestimate their performance. Subjects overestimated their performance at 50% but underestimated their performance at 85% intelligibility level. In line with our previous study [14], this bias partly explains the association between the lower performance ratings and high effort ratings. In other words, both higher subjective effort ratings and lower subjective performance ratings related to a larger PPD. One of the advantages of PPD over the subjective effort ratings is the immediacy of the measurements. Instead of providing a global subjective score at the end of a block, PPD was measured during each sentence. It is also insensitive to response biases. In all, these results are in line with the idea that listening effort relates to cognitive load as measured by pupillometry. 

Finally, the amount of variance accounted for in the models predicting cognitive load, was smaller than the variance explained in the models predicting SRT. This may indicate that the top-down functions captured by the cognitive tests used in our study are more relevant for explaining performance than for processing load. This leaves us with the question as to what top-down functions or other individual factors may be used to solve “high-load” conditions. Attention could be such a factor [25] and this deserves further investigation in future research. In addition, the variance in the models explaining processing load was not accounted for by WMC. It has often been suggested that the influence of WMC in speech comprehension is mainly used to solve processing under adverse conditions [15, 35–37]. The current study demonstrated that the TRT test (linguistic abilities) [3, 19, 20] accounted for part of the variance as well as the ability to inhibit irrelevant speech [17], rather than WMC. This outcome illustrates that more research is needed to find out all processes responsible for cognitive load during speech processing in adverse listening conditions. Pupillometry seems a fitting method because it already revealed a number of insights that could not have been shown by traditional outcome measures like speech intelligibility scores and subjective ratings. 

Traditionally, speech performance scores are used to evaluate the benefit of hearing aid amplification, but an urgent question is whether hearing aids are also able to reduce the listening effort people experience in daily-life listening. Pupillometry is a promising method, which may provide us with additional insight in the benefit of hearing aids. For people with hearing impairment, recognition of speech in background sound is more challenging than for normal hearing people. This might explain the higher levels of listening effort and fatigue as reported by people with hearing loss [3, 53]. A logical next research step would be to investigate the effect of hearing impairment and hearing aids on cognitive load during speech processing. The influential studies by Gatehouse et al. [12, 13] showed that the amount of benefit people derive from specific types of amplification is related to their cognitive capacity. Unfortunately, these benefits were only investigated and observed at the level of speech recognition. The current method of pupillometry is a promising method to additionally test for the effects of aided versus unaided listening on listening effort. Taking into account the effects of a hearing aid on listening effort as represented by PPDs would possibly bring hearing aid fitting a substantial step forward.

## 5. Conclusions 

People with better cognitive abilities show lower signal-to-noise ratios for speech perception at fixed performance levels (50 or 84%), indicating that they were better able to ignore the noise. At the same time, those with better abilities to ignore the noise exploited slightly more processing load. This effect becomes most prominent when speech is masked by speech uttered by an interfering talker. It is expected that the ability to ignore irrelevant information during speech communication and the related processing load is also an important factor determining hearing aid benefit. One of the advantages of pupillometry is the immediacy of the measurements and as such it is also a promising method for the evaluation of hearing aid benefit. Future research should investigate the association between aided listening, cognitive capacities, and listening effort.

## Figures and Tables

**Table 1:** The average SRT scores, PPD, and the subjective ratings for both levels of intelligibility and for both masker types.

Intelligibility	Fluctuating	Single talker
SRT	SNR (SD), dB	
50%	−11.2 (1.5)	−12.6 (1.9)
84%	−5.7 (1.5)	−6.0 (2.8)

Pupil	PPD (SD), mm	
50%	0.23 (.16)	0.29 (.16)
84%	0.16 (.12)	0.22 (.16)

Subjective	Effort (low = 0–high = 10)	
50%	6.9 (1.5)	6.7 (1.3)
84%	4.7 (1.7)	5.3 (1.6)
	Performance (low = 0–high = 10)	
50%	5.5 (1.4)	5.4 (1.0)
84%	7.1 (1.0)	6.9 (1.2)
	Motivation (low = 0–high = 10)	
50%	8.0 (1.4)	7.9 (1.7)
84%	8.6 (1.3)	8.6 (1.3)

**Table 2:** Two-tailed Pearson correlations (*P < .05, **P < .01) between age, PTA, Rspan, Lspan, SICspan, TRT, SRT with fluctuating noise at 50% (SRT_F50_) and 84% (SRT_F84_) intelligibility, and SRT with a single-talker masker at 50% (SRT_ST50_) and 84% (SRT_ST84_) intelligibility. Lower TRTs and SRTs indicate better performance.

	Age	PTA	Rspan	Lspan	SICspan	TRT
Age	X					
PTA	.468**	X				
Rspan	−.299	−.316	X			
Lspan	−.048	−.182	.669**	X		
SICspan	−.278	−.390*	.658**	.585**	X	
TRT	.305	.330	−.759**	−.584**	−.684**	X
SRT_F50_	.342	.097	−.079	−.317	−.208	.186
SRT_F84_	.235	.105	−.361*	−.261	−.430*	.248
SRT_ST50_	.352*	.203	−.501**	−.348	−.480**	.673**
SRT_ST84_	.509**	.284	−.463**	−.282	−.499**	.540**

**Table:** (a)

Fluctuating	SRT_F50_			SRT_F84_		
	*B*	*R* ^2^	*P*	*B*	*R* ^2^	*P*
Rspan	−.03	.01	.668	−.11*	.15	.086
Lspan	−.14	.10	.077	−.11	.07	.149
SICspan	−.05	.04	.254	−.11	.19	.014
TRT	.06	.04	.308	.08	.06	.171

Single talker	SRT_ST50_			SRT_ST84_		
	*B*	*R* ^2^	*P*	*B*	*R* ^2^	*P*

Rspan	−.19*	.30	.013	−.22*	.37	.036
Lspan	−.18	.121	.051	−.22	.08	.118
SICspan	−.13*	.28	.017	−.18*	.40	.015
TRT	.29	.45	.000	.27*	.42	.008

**Table:** (b)

Fluctuating	PPD_F50_			PPD_F84_		
	*B*	*R* ^2^	*P*	*B*	*R* ^2^	*P*
Rspan	.01	.03	.315	.00	.01	.650
Lspan	.00	.00	.744	.00	.00	.994
SICspan	.01	.08	.109	.01	.08	.107
TRT	−.01	.10	.075	−.01	.05	.220

Single talker	PPD_ST50_			PPD_ST84_		
	*B*	*R* ^2^	*P*	*B*	*R* ^2^	*P*

Rspan	.01	.05	.208	.01	.04	.282
Lspan	.00	.00	.813	.00	.01	.680
SICspan	.01	.13	.047	.01	.13	.040
TRT	−.02	.19	.014	−.01	.12	.056

**Table 4:** Associations (*P* < .05) between the dependent variables Δ_SRT_ and Δ_PPD_ [Δ = (ST50 + ST84/2) − (F50 + F84/2)], and the cognitive capacity measures. Shown are the unstandardized regression coefficients (*B*) and the variance (*R* ^2^). In none of the analyses PTA appeared to be a significant confounder. We adjusted for age (*) in the models in which age was a significant confounder.

	Δ_SRT_			Δ_PPD_		
	*B*	*R* ^2^	*P*	*B*	*R* ^2^	*P*
Rspan	−.15*	.17	.085	.00	.07	.144
Lspan	−.08	.02	.462	.00	.01	.636
SICspan	−.11	.10	.077	.00	.09	.090
TRT	.24	.25	.003	−.01	.18	.017
